# Investigating the impact of persistent HPV infection on recurrence of lesions post-surgery for early-stage cervical cancer and related influencing factors

**DOI:** 10.3389/fonc.2025.1506521

**Published:** 2025-02-04

**Authors:** Jing Na, Ya Li, Qiao Lu, Yang Wang, Shichao Han, Jun Wang

**Affiliations:** Department of Gynecology and Obstetrics, Second Affiliated Hospital of Dalian Medical University, Dalian, China

**Keywords:** HPV infections, cervical carcinoma, hysterectomy, recurrence, HPV - human papillomavirus

## Abstract

**Objective:**

To explore the influencing factors of recurrence after surgical treatment for early-stage cervical cancer (stages IA1-IIA1) and to investigate the relationship between human papillomavirus (HPV) infection and postoperative recurrence of lesions.

**Methods:**

A retrospective analysis was conducted on the clinical data of 242 patients who underwent surgical treatment for early-stage cervical cancer (FIGO stages IA1-IIA1) at the Second Affiliated Hospital of Dalian Medical University between 2015 and 2022. Cox regression analysis was employed to evaluate the relationship between persistent postoperative HPV infection and lesion vaginal local recurrence while identifying the associated risk factors for persistent HPV infection following surgery.

**Results:**

Within 12 months postoperatively, the HPV clearance rate was 88.11%. HPV infection persisted beyond 12 months in 19 patients (7.9%), with 3 cases demonstrating the same HPV genotypes (types 52, 58) as those identified preoperatively. Multivariate analysis identified persistent postoperative HPV infection (odds ratio [OR] 1.72, 95% confidence interval [CI] -1.14 to 5, p=0.001*) as an independent risk factor for recurrence. Additionally, smoking (OR 7.49, 95% CI 1.19 to 47.13, p=0.032), abnormal vaginal microbiota (OR 0.663, 95% CI 0.403 to 1.088, p=0.001*), and the type of surgical procedure (OR 0.32, 95% CI 0.11 to 0.91, p=0.033) were significantly associated with a higher rate of persistent HPV infection.

**Conclusion:**

Persistent HPV infection after surgery is an independent risk factor for postoperative recurrence in early-stage cervical cancer. Surgical approach, abnormal vaginal microbiota, and smoking are associated factors for persistent HPV infection after surgery.

## Introduction

Cervical cancer is one of the most prevalent malignancies affecting women globally, with an increasing incidence trend (1). Surgical intervention is the standard treatment for early-stage cervical cancer (2). Postoperative recurrence remains a leading cause of mortality in cervical cancer patients, underscoring the importance of vigilant follow-up and early detection in preventing recurrence. Numerous studies have identified postoperative pathological factors, including tumor size, depth of stromal invasion, lymphovascular space invasion (LVSI), pathologically confirmed lymph node metastasis, parametrial involvement, and positive surgical margins, as significant predictors of recurrence. Adjuvant chemoradiotherapy is recommended for patients with these pathological risk factors following surgery. With the continuous advancement of cervical cancer screening programs, an increasing number of cases are being diagnosed at an early stage. Thus, exploring the factors associated with postoperative recurrence in early-stage cervical cancer is of considerable clinical importance for optimizing postoperative surveillance and improving risk assessment for recurrence. Numerous studies have identified human papillomavirus (HPV), particularly high-risk strains such as HPV16 and HPV18, as the primary etiological factor in the onset and progression of cervical cancer (2, 3). Persistent infection with high-risk HPV can induce cervical epithelial lesions, which may ultimately progress to invasive cervical carcinoma (3). This study investigated the risk factors for lesion vaginal local recurrence in 242 patients with early-stage cervical cancer following surgical treatment. Furthermore, it examined the association between persistent HPV infection and lesion vaginal local recurrence, as well as the high-risk factors contributing to persistent HPV infection. The objective is to provide a foundation for the prevention and management of postoperative recurrence in cervical cancer and to offer scientific evidence for the development of personalized follow-up and management strategies.

## Methods

This study employed a retrospective analysis, collecting clinical data from 242 patients with early-stage cervical cancer treated at the Second Affiliated Hospital of Dalian Medical University between 2015 and 2022. The data included (1): Demographic and medical history, such as age, gravidity and parity, smoking history, and immune status (2); Preoperative and postoperative HPV test results (3); Surgical information, including the type of procedure and postoperative pathological findings; and (4) Postoperative follow-up data, including HPV test results at 3, 6, 12, and 24 months post-surgery, vaginal microecological status, sexual activity, and recurrence of lesions. The inclusion criteria were (1): FIGO stage I-IIA1 cervical cancer, with patients undergoing extrafascial hysterectomy or radical hysterectomy (2); Patients who provided informed consent for TCT and HPV testing; and (3) Patients who agreed to regular follow-up. Exclusion criteria (1): Patients lost to follow-up or those who did not complete follow-up (2); Patients with severe liver, kidney, or lung diseases (3); Patients with other malignant tumors of the reproductive system (4); Patients on long-term immunosuppressive therapy. Elimination criteria (1): Patients with missing data (2); Patients unable to undergo sampling or those presenting with symptoms such as vaginal bleeding or discharge. Follow-up content: By reviewing patients’ outpatient and inpatient medical records, as well as relevant lab results and examinations, data were collected on patients’ age, smoking history, sexual activity, pathological type, and vaginal secretions. HPV testing and clearance evaluation: HPV sampling was conducted for all patients during follow-up visits post-surgery. Statistical analysis: Data were processed using SPSS statistical software. Continuous variables were presented as mean ± standard deviation, and differences between groups were evaluated using one-way analysis of variance (ANOVA). The Kaplan-Meier method was utilized for survival analysis to compare differences between groups, while Spearman’s rank correlation was applied to assess correlations. Univariate analysis was conducted to identify factors influencing postoperative recurrence, and significant variables were subsequently included in a multivariate Cox regression analysis to determine independent risk factors for recurrence. A t-test with a significance level of α=0.05 was employed, with p<0.05 considered statistically significant.

## Results

### Characteristics of patients

A retrospective analysis was performed on the clinical data of patients who underwent surgical treatment for early-stage cervical cancer (FIGO stages IA1-IIA1) at the Second Affiliated Hospital of Dalian Medical University between 2015 and 2022, with 242 patients meeting the inclusion criteria. According to FIGO staging, extrafascial hysterectomy or radical hysterectomy combined with lymphadenectomy was selected. Patients with pathological risk factors additionally received adjuvant chemoradiotherapy postoperatively. The mean age of the 242 patients was 52 years (range: 25–76 years). Pathological evaluation confirmed that none of the patients had parametrial involvement or positive surgical margins. Of the 242 patients, preoperative HPV tests were positive in all, with 203 cases (83.8%) infected with HPV-16/18, and 34 cases (14.1%) infected with other high-risk HPV (HR-HPV) genotypes. The baseline characteristics of the patients are shown in **Table 1**.

**Table 1:** Patient’s baseline data.

Variables		Value
Age (year)		52.10 ± 10.22
BMI (kg/m^2^)		23.93 ± 4.11
SCC (ng/ml)		2.71 ± 4.60
Menopause	Yes	139
	No	103
Smoking	Yes	9
	No	233
Surgical approach	Minimally invasive	117
	Laparotomy	125
Pathological type	Squamous carcinoma	206
	Adenocarcinoma	30
	Adenosquamous Carcinoma	6
LVSI	Yes	53
	No	189
Tumor differentiation	**Poorly**	26
	Moderately	146
	Well	70
FIGO stage	IA1	67
	IA2	27
	IB1	89
	IB2	37
	IIA1	22
Vaginal microecology	Normal	212
	Disorder	30

Postoperative pathological results for all patients showed no lymph node metastasis, and the postoperative FIGO staging of the patients remained unchanged.

All patients underwent HPV testing at intervals of 3 to 12 months postoperatively. In this study, the duration of postoperative HPV infection was defined as the time from the date of surgery to the detection of HPV infection, with the first negative conversion observed following cytological examination of exfoliated cells from the vaginal cuff during follow-up. A total of 21 patients had persistent HPV infection after surgery, with 2 cases clearing within 12 months, 11 cases clearing between 12 and 24 months, and 8 cases remaining HPV-positive beyond 24 months. Eight cases of postoperative recurrence-related lesions were observed: four cases of tumor recurrence (two cases of adenocarcinoma and two cases of squamous cell carcinoma); and four cases of secondary lesions, with vaginal cuff biopsy indicating squamous intraepithelial lesions.

### Analysis of influencing factors of recurrence

In univariate analysis, persistent postoperative HPV infection was significantly associated with an increased risk of local recurrence. Multivariate Cox regression analysis further identified persistent HPV infection after surgery (odds ratio [OR] 1.72, 95% confidence interval [CI] -1.14 to 5, p=0.001*) as an independent risk factor for local recurrence (**Table 2**).

**Table 2:** Univariate analysis and multivariate analysis to evaluate the influencing factors of local recurrence.

Variables	Univariate analysis (p-value)					Multivariate analysis(p-value)				
	β	S.E	Z	*P*	OR (95%CI)	β	S.E	Z	*P*	OR (95%CI)
Age	0.01	0.03	0.37	0.711	1.01 (0.95 ~ 1.08)					
BMI	-0.10	0.11	-0.89	0.372	0.91 (0.73 ~ 1.12)					
Gravidity	0.05	0.23	0.22	0.822	1.05 (0.67 ~ 1.64)					
Parity	-0.54	0.54	-0.99	0.321	0.58 (0.20 ~ 1.69)					
SCC	-0.08	0.16	-0.52	0.602	0.92 (0.68 ~ 1.25)					
Menopause	0.39	0.72	0.54	0.591	1.47 (0.36 ~ 6.04)					
Smoking	-14.38	1495.30	-0.01	0.992	0.00 (0.00 ~ Inf)					
Surgical approach	-1.43	0.81	-1.76	0.078	0.24 (0.05 ~ 1.17)	-1.97	0.85	-2.32	0.020*	0.14 (0.03 ~ 0.74)
Pathological type	0.99	0.53	1.86	0.063	2.68 (0.95 ~ 7.61)	0.68	0.55	1.24	0.216	1.98 (0.67 ~ 5.82)
LVSI	0.55	0.73	0.76	0.445	1.74 (0.42 ~ 7.21)					
Tumor differentiation	-0.70	0.60	-1.17	0.243	0.49 (0.15 ~ 1.61)					
FIGO stage	-0.48	0.29	-1.63	0.104	0.62 (0.35 ~ 1.10)	-0.68	0.32	-2.14	0.032*	0.51 (0.27 ~ 0.94)
Postoperative HPV	-2.882	0.98	-1.109	0.001*	0.60(-21.01~-1.51)	-2.81	0.36	1.737	0.001*	1.72(-1.14~5)

*P<0.05; LVSI, lymphovascular space invasion.

### Analysis of influencing factors of HPV Infection

In univariate analysis, smoking and abnormal vaginal microecology were found to be associated with an increased incidence of persistent HPV infection. Multivariate Cox regression analysis identified the surgical approach and smoking as significant risk factors for persistent HPV infection (**Table 3**).

**Table 3:** Univariate analysis and multivariate analysis to evaluate the influencing factors of HPV Infection.

Variables	Univariate analysis (p-value)					Multivariate analysis(p-value)				
	β	S.E	Z	*P*	OR (95%CI)	β	S.E	Z	*P*	OR (95%CI)
Age	0.01	0.02	0.62	0.536	1.01 (0.97 ~ 1.06)					
BMI	0.01	0.07	0.10	0.919	1.01 (0.88 ~ 1.16)					
Gravidity	0.15	0.16	0.98	0.325	1.17 (0.86 ~ 1.58)					
Parity	0.44	0.27	1.62	0.106	1.55 (0.91 ~ 2.65)	0.51	0.27	1.86	0.063	1.66 (0.97 ~ 2.83)
SCC	-0.21	0.17	-1.19	0.233	0.81 (0.58 ~ 1.14)					
Menopause	0.25	0.50	0.50	0.616	1.28 (0.49 ~ 3.38)					
Smoking	1.60	0.87	1.83	0.068	4.94 (0.89 ~ 27.39)	2.01	0.94	2.15	0.032*	7.49 (1.19 ~ 47.13)
Surgical approach	-0.97	0.51	-1.89	0.058	0.38 (0.14 ~ 1.04)	-1.13	0.53	-2.14	0.033*	0.32 (0.11 ~ 0.91)
Pathological type	-0.05	0.58	-0.09	0.925	0.95 (0.31 ~ 2.93)					
LVSI	-0.10	0.59	-0.17	0.862	0.90 (0.29 ~ 2.85)					
Tumor differentiation	-0.39	0.42	-0.93	0.353	0.68 (0.30 ~ 1.54)					
FIGO stage	-0.11	0.16	-0.67	0.504	0.90 (0.65 ~ 1.24)					
Abnormal vaginal microecology	1.844	0.014	0.193	0.001*	0.663 (0.403~1.088)	-0.61	0.43	1.20	0.091	4.63 (1.20~19.979)

*P<0.05; LVSI, lymphovascular space invasion.

## Discussion

Surgical intervention remains the principal approach for the management of early-stage cervical cancer; however, a subset of patients may still encounter postoperative recurrence. Research has demonstrated that the recurrence rate for cervical cancer can reach up to 13.40% several years post-surgery (4). Research has demonstrated that the prognosis of early-stage cervical cancer is closely linked to various pathological risk factors, such as lymph node metastasis, positive surgical margins, tumor size, parametrial invasion, deep stromal invasion, and lymphovascular space invasion (LVSI) (5). This study found that the risk of postoperative recurrence is increased for the pathological type of adenocarcinoma. Beriwal S et al. (6) observed that the five-year survival rate for adenocarcinoma is lower compared to that of squamous cell carcinoma. The elevated recurrence rate associated with adenocarcinoma may be attributed to its propensity for deep muscular layer invasion and the challenges in early detection, coupled with a heightened risk of lymph node metastasis. Furthermore, adenocarcinoma tends to exhibit reduced sensitivity to radiation therapy, which constrains available treatment options. Several studies also indicate that pathological type serves as an independent risk factor for the prognosis of cervical cancer patients (7). Jung et al. (8) discovered that cervical cancer patients who underwent radical surgery for squamous cell carcinoma exhibited better prognoses compared to their counterparts with adenocarcinoma, evidenced by a higher five-year survival rate for squamous cell carcinoma. This finding aligns with the results of prior studies. Consequently, it is imperative to prioritize and enhance long-term follow-up for patients diagnosed with adenocarcinoma post-surgery.

Beyond pathological risk factors, research exploring the relationship between HPV infection and postoperative prognosis in cervical cancer has attracted considerable attention, particularly regarding aspects such as HPV infection status and genotype. The HPV status following surgery for early-stage cervical cancer represents a critical factor in the postoperative follow-up management of cervical cancer patients. It is widely acknowledged that persistent HPV positivity may signify an elevated risk of recurrence. Consequently, elucidating the relationship between HPV status and recurrence is essential for effective clinical management and accurate prognosis assessment (9–11). HPV status remains an important area of research in postoperative prognostic assessment. In patients with early-stage cervical cancer, HPV infection status is considered one of the key factors influencing postoperative prognosis. Studies have shown that HPV-positive patients typically have poorer survival rates and higher recurrence risks. In cervical cancer patients who undergo radical surgery, high-risk HPV infection is significantly associated with adverse prognosis (12). For patients who remain positive for high-risk HPV after surgery, the risk of recurrence is significantly increased, and regular monitoring of their viral status is necessary postoperatively (13). This study found that persistent HPV infection following surgery for early-stage cervical cancer significantly elevates the risk of lesion vaginal local recurrence and acts as an independent risk factor for disease relapse. Following surgical treatment for early-stage cervical cancer, HPV infection generally resolves as the lesions are eradicated. However, some patients persistently exhibit HPV infection status post-surgery. Research conducted by Kreimer et al. (14) demonstrates a close association between postoperative persistent HPV positivity and cervical cancer recurrence. Song Dan (15) found that persistent HPV infection following cervical cancer surgery is significantly associated with pelvic recurrence, whereas no significant correlation was identified with distant metastasis. Persistent high-risk HPV infection elevates the risk of pelvic recurrence without influencing distant metastasis, potentially due to differences in the underlying mechanisms of pelvic recurrence and distant metastasis. Postoperative persistent HPV positivity in cervical cancer may suggest the presence of potential residual lesions, particularly in cases with unclear surgical margins or significant tumor infiltration depth. These residual lesions may remain susceptible to HPV, potentially resulting in recurrent lesions or the emergence of new disease foci. Multiple studies have shown that persistent HR-HPV infection after cervical cancer treatment is significantly associated with disease recurrence (15, 16). Although several studies have explored HPV as a factor in the postoperative prognosis of early-stage cervical cancer, more large-scale clinical trials are needed to validate these findings and to investigate the specific effects of different types and subtypes of HPV on treatment response and long-term survival rates. This will help in developing personalized treatment plans, improving the overall survival rates and quality of life for early-stage cervical cancer patients. Current research indicates that, although cervical cancer can be effectively prevented through vaccination and screening measures, the incidence of cervical cancer remains high in many regions, particularly in low-income countries (17). Early-stage cervical cancer is typically treated through surgery, including radical hysterectomy and pelvic lymphadenectomy. However, there is still debate over whether patients should receive the HPV vaccine postoperatively to further reduce the risk of recurrence and improve survival rates. Some studies suggest that vaccination with the HPV vaccine can significantly reduce the incidence of tumors related to HPV types not previously infected and may provide additional protection to patients who have already undergone treatment (18). However, there is currently a lack of systematic research on the specific effects of HPV vaccination and the optimal timing for vaccination in patients with early-stage cervical cancer after surgery. Some studies suggest that in women who have undergone radical surgery, receiving the HPV vaccine can enhance the immune response, potentially reducing the risk of recurrence (19). Although existing studies support the use of HPV vaccination in early-stage cervical cancer patients after surgery to reduce the risk of recurrence, more randomized controlled trials are needed to verify the effectiveness and safety of this strategy.

As an increasing number of studies suggest, effective monitoring of HPV infection following initial standardized treatment serves as a crucial predictor of recurrence (20, 21).

In summary, although the surgical treatment has removed the lesions, the persistent infection of HPV, particularly HR-HPV, after surgery indicates that the carcinogenic factors have not been fully eradicated. While local recurrence of the tumor may be associated with the metastatic properties of the tumor itself, the combination of persistent HPV infection post-surgery and the characteristics of tumor recurrence significantly increases the recurrence rate.

This study identified smoking and abnormal vaginal microbiota as significant risk factors for persistent HPV infection following surgery for early-stage cervical cancer. In the majority of early-stage cervical cancer patients, HPV infection is likely to resolve naturally within 6 to 12 months after radical surgery. Among the 242 patients examined in this study, HPV infection initially exhibited a rapid resolution trend, followed by a gradual decline, with the peak period for achieving HPV negativity occurring within 12 months post-surgery. Research has indicated that a sustained increase in HPV-DNA levels within 12 months post-surgery is an independent risk factor for higher local recurrence (21). Based on the patterns of HPV regression following cervical cancer surgery, it is essential to enhance monitoring during early postoperative follow-up and implement appropriate intervention measures when necessary, as these actions are critical for prolonging patient survival and improving prognosis. The vaginal microbiota constitutes a complex microenvironment comprising diverse bacterial communities that play a vital role in preserving vaginal health, preventing infections, and supporting immune function. The persistent presence or recurrence of HPV infection following cervical cancer surgery is closely associated with the patient’s immune status, while an imbalance in the vaginal microbiota may serve as a significant factor affecting the clearance of HPV postoperatively and contributing to persistent infection. The normal vaginal microbiota is predominantly composed of lactobacilli (such as Lactobacillus species), which help maintain the vagina’s acidic environment, typically characterized by a pH range of 3.8 to 4.5, through the production of lactic acid. This acidic milieu inhibits the proliferation of pathogenic microorganisms and prevents the infection and dissemination of viruses such as HPV via competitive mechanisms. When the balance of the vaginal microbiota is disrupted, a decline in lactobacilli can occur, resulting in an overgrowth of pathogenic bacteria (such as Gardnerella and anaerobic bacteria). This shift leads to an elevation in vaginal pH, which subsequently compromises the natural barrier function against viruses (22–24). This study identified abnormal vaginal microbiota as a significant risk factor for persistent HPV infection in patients with early-stage cervical cancer following surgery (P < 0.05).Research by Bober et al. (25) demonstrates that the vaginal microbiota not only modulates HPV infection but is also linked to the rate of viral clearance after HPV infection and the risk of malignant transformation. Persistent HPV infection can disrupt the vaginal microbiota, resulting in the suppression of immune function in vaginal mucosal cells. Various microbial communities colonizing the surface of the vaginal epithelial mucosa interact with one another to maintain the vaginal microbiota in a relatively balanced state. However, this equilibrium is inherently unstable and can often be disrupted or dysregulated by factors such as decreased estrogen levels, inappropriate antibiotic use, and compromised immune function. As dysbiosis in the vaginal microbiota intensifies, the risk of persistent HPV infection correspondingly rises. Consequently, genital infections and imbalances in the vaginal microbiota significantly contribute to the development of cervical cancer and the recurrence of the disease following surgical intervention.

This study found no correlation between extrafascial hysterectomy and persistent HPV infection in the univariate analysis. However, multivariate Cox regression analysis demonstrated that the risk of persistent HPV infection is significantly elevated following extrafascial hysterectomy, thereby identifying it as a potential risk factor. Drawing upon the theory of differentiation of various embryonic units of pelvic organs, the female reproductive tract is associated with the paramesonephric duct (commonly referred to as the Müllerian duct) and the urogenital sinus embryonic units during embryonic development (26). By the ninth week of embryonic development, the cranial portions of the bilateral paramesonephric ducts develop into the fallopian tubes, while the middle and caudal segments fuse at the midline to form the uterine body, cervix, and upper portion of the vaginal fornix. The tissue origins of the upper and lower segments of the vagina differ: the upper segment arises from the paramesonephric duct, whereas the lower segment is derived from the urogenital sinus. Extrafascial hysterectomy is categorized as the excision of non-embryonic unit organs and is typically indicated for patients with early local dissemination. This procedure facilitates the comprehensive removal of affected tissue; however, some potential sites of HPV infection may still persist. As a result, the incidence of persistent HPV infection following extrafascial hysterectomy is higher compared to that after radical hysterectomy, while the rate of persistent HPV infection after hysterectomy remains lower than that observed following cone biopsy (27). In specific situations, if the surgery fails to completely excise all regions of HPV infection or if the patient’s postoperative immune response is compromised, HPV may continue to persist within the local tissues. As a result, the incidence of persistent HPV infection following surgery may rise. Chen et al. (21) discovered that women with viral loads ≥5.22 copies/10^4 cells are at an increased risk of residual lesions. Furthermore, they observed significant variations in the quantity of residual lesions correlated with HPV-16/52/58 viral loads, in contrast to HPV-31/33. This indicates that high-risk HPV (HR-HPV) viral load may serve as a reliable predictor for the presence of residual lesions. The SHAPE study (28), presented at the 2023 American Society of Clinical Oncology (ASCO) annual meeting, demonstrated that simple total hysterectomy is comparable to radical hysterectomy in the management of early-stage cervical cancer. Furthermore, the study found no significant statistical differences in relapse-free survival (RFS) and overall survival (OS) rates between the two surgical approaches. A meta-analysis demonstrated that for patients with stage IA2 to IB1 cervical cancer, there were no statistically significant differences in recurrence rates or overall survival between those who underwent extraperitoneal hysterectomy and those who underwent radical hysterectomy (29). Therefore, a comprehensive analysis of the correlation between postoperative prognosis and surgical methods for early cervical cancer should consider multiple factors. Persistent HPV infection following surgery may be a significant contributor to prognosis. Consequently, it is crucial to implement enhanced monitoring and follow-up after surgery, and to intensify adjuvant therapy when necessary, in order to optimize patient outcomes.

## Conclusion

This study demonstrates that persistent HPV infection following surgery serves as an independent risk factor for local recurrence in early cervical cancer. Additionally, abnormal vaginal discharge, surgical approach, and smoking are identified as contributing risk factors for persistent HPV infection post-surgery. Therefore, early and accurate HPV testing following surgery, combined with the monitoring of associated influencing factors, is instrumental in assessing the risk of postoperative recurrence in patients. This strategy can facilitate more effective postoperative management and intervention approaches, ultimately enhancing both the cure rate and quality of life for patients with early cervical cancer.

## Data Availability

The original contributions presented in the study are included in the article/supplementary material. Further inquiries can be directed to the corresponding authors.
